# Alleviation of banded leaf and sheath blight disease incidence in maize by bacterial volatile organic compounds and molecular docking of targeted inhibitors in *Rhizoctonia solani*

**DOI:** 10.3389/fpls.2023.1218615

**Published:** 2023-09-13

**Authors:** Humaira Yasmin, Zafar Abbas Shah, Saqib Mumtaz, Noshin Ilyas, Urooj Rashid, Abdulaziz Abdullah Alsahli, Yong Suk Chung

**Affiliations:** ^1^ Department of Biosciences, COMSATS University Islamabad (CUI), Islamabad, Pakistan; ^2^ Department of Bioinformatics, Hazara University, Mansehra, Pakistan; ^3^ Department of Botany, PMAS Arid Agriculture University, Rawalpindi, Pakistan; ^4^ Botany and Microbiology Department, College of Science, King Saud University, Riyadh, Saudi Arabia; ^5^ Department of Plant Resources and Environment, Jeju National University, Jeju, Republic of Korea

**Keywords:** volatile organic compounds (VOCs), 2-pentylfuran, 2,3-butanediol, dimethyl disulfide, docking, CRZ1 receptor, S9 receptor, banded leaf and sheath blight, Rhizoctonia solani

## Abstract

*Rhizoctonia solani* (RS) is a pathogenic fungus that affects maize (*Zea mays* L.) plants and causes banded leaf and sheath blight (BLSB) with severe consequences leading to significant economic losses. Contrarily, rhizobacteria produce numerous volatile organic compounds (VOCs) that help in devising the environment-friendly mechanism for promoting plant growth and stress alleviation without having physical contact with plants. In the present study, 15 rhizobacterial strains were tested for their antagonism against RS. The antagonistic potential of VOCs of the tested plant growth-promoting rhizobacteria (PGPR) strains ranged from 50% to 80% as compared to the control (without PGPR). Among these 15 strains, the maximum (80%) antagonistic activity was exhibited by *Pseudomonas pseudoalcaligenes* SRM-16. Thus, the potential of VOCs produced by *P. pseudoalcaligenes* SRM-16 to alleviate the BLSB disease in maize was evaluated. A pot experiment was conducted under greenhouse conditions to observe the effect of VOCs on disease resistance of BLSB-infected seedlings. Overall, maize seedlings exposed to VOCs showed a significant increase in disease resistance as indicated by a reduced disease score than that of unexposed infected plants. The VOCs-exposed maize exhibited lower (11.6%) disease incidence compared to the non-inoculated maize (14.1%). Moreover, plants exposed to VOCs displayed visible improvements in biomass, photosynthetic pigments, osmoregulation, and plant antioxidant and defense enzyme activities compared to the healthy but unexposed seedlings. Simultaneous application of RS and VOCs enhanced superoxide dismutase (SOD), peroxidase (POD), catalase (CAT), phenylalanine ammonia lyase (PAL), ascorbate peroxidase (APX), and polyphenol oxidase (PPO) activities by 96.7%, 266.6%, 313.7%, 246.6%, 307%, and 149.7%, respectively, in the roots and by 81.6%, 246.4%, 269.5%, 269.6%, 329%, and 137.6%, respectively, in the shoots, relative to those of the control plants. The binding affinity of the VOCs (2-pentylfuran, 2,3-butanediol, and dimethyl disulfide) with CRZ1 and S9 protein receptors of RS was assessed by deploying *in silico* methods. Overall, 2-pentylfuran exhibited a binding affinity with both the selected receptors of RS, while 2,3-butanediol and dimethyl disulfide were able to bind S9 protein only. Hence, it can be deduced that S9 protein receptors are more likely the target RS receptors of bacterial VOCs to inhibit the proliferation of RS.

## Introduction

Worldwide, approximately 9.4% of maize is annually lost to infections caused by pathogenic viruses, bacteria, and fungi (Munkvold et al., 2019). Common fungal diseases of maize include bacterial leaf spot, stalk rot, brown spot, charcoal rot, gray leaf spot, and banded leaf and sheath blight (BLSB) (Mueller et al., 2016; Qiu et al., 2020). Among these, BLSB is considered a serious disease that manifests initially on leaves and sheaths of the maize as leaf sheath blight and in the later stages spread to the ears as ear rot. The disease has been reported in the United States, Germany, England, Venezuela, Nigeria, Ivory Coast, and Sierra Leone. Particularly, BLSB is considered as a serious threat to maize yield in China, South Asia, and Southeast Asia (India, Indonesia, Sri Lanka, Cambodia, Pakistan, Bangladesh, Nepal, Myanmar, Thailand, Vietnam, Laos, Philippines, Malaysia, Taiwan, Japan, and Korea). Strikingly, in China, up to 100% yield losses have been attributed to this disease (Hooda et al., 2017). BLSB is incited by the fungus *Rhizoctonia solani* and it infects maize during hot and humid weather (Li et al., 2019). As it is a soil-borne disease, the pathogen starts spreading from the basal sheath to the developing ear, completely damaging the ear and caking of husk leaves that dry off and fall (Li et al., 2019). *Rhizoctonia solani* is a necrotroph that releases phytotoxins in the course of infection, causing necrotic spots on the leaf, sheath, and stem. Under favorable conditions, it infects nearly all aerial parts of the plants including cobs and tassels. Heavily infected cobs may not produce grains and resting spores (sclerotia) may form inside the cobs (Singh et al., 2020). The grain yield loss by the disease ranges from 11% to 40% reaching up to 100% in some highly humid and warm regions where conducive conditions prevail for the pathogen (Hooda et al., 2017).

The suppression of persistent pathogen spores from the soil is challenging for farmers. To mitigate biotic stresses in plants, various strategies like sterilization of plowing machinery, chemical fungicides, and soil fumigators have been devised and adopted as regular practices (Singh et al., 2020). However, sterilization of plowing machinery for each field is not feasible, while chemical fungicides are ecologically unfriendly, very expensive, and responsible for the transfer of drug resistance to human fungal pathogens. However, shortcomings of the abovementioned techniques can be minimized by exploiting plant growth-promoting rhizobacteria (PGPR) as an efficient, economic, environment-friendly, and sustainable disease management alternative to enhance long-term plant defense responses.

There is a considerable amount of literature addressing the direct and indirect role of PGPR for biocontrol activity and nutrient management for improved plant growth (Singh et al., 2020; Yasmin et al., 2020b). Plant growth-promoting rhizobacteria have been found to act as a biocontrol agent directly by killing the pathogen by having close physical contact while unaccompanied by the support of the microbial community and immediate surroundings (Tilocca et al., 2020). In contrast, competition and systemic resistance of hosts are indirect modes of antagonism that involve the production of metabolites that make the surroundings adverse for the survival of pathogens (Tilocca et al., 2020). Moreover, PGPR revamp plant defense mechanisms under pathogen stresses by altering the genetic, biochemical, and morphological responses of plants (Jha, 2018). They also operate by stimulating various defense compounds during pathogen attacks. It has been found that bacteria are associated with the observed biocontrol activities against plant pathogens by induced systemic resistance (Jha, 2018).

Mostly, root colonization of PGPR is believed to be the most important factor to certainly stimulate plant growth by modulating development, harvest, and disease resistance (Amna et al., 2020; Tilocca et al., 2020; Yasmin et al., 2020a). However, understanding the possible mechanism of rhizobacteria to mediate plant responses to external stimuli (biotic and abiotic stresses) by releasing volatile signals (Tilocca et al., 2020) has been gaining interest recently. One group of these signals is usually volatile organic compounds (VOCs) that are produced as bacterial secondary metabolites (Rath et al., 2018). They are characterized by low molecular weight, low boiling point, odorous nature, lipophilic moiety, and high vapor pressure (Schulz-Bohm et al., 2017). Volatile organic compounds are species-specific and also alter following the growth conditions of the microorganisms (Almeida et al., 2023). Researchers have reviewed bacterial species and their VOCs and antagonistic activities against different plant pathogens (Bukhat et al., 2020).

Owing to the abovementioned properties of VOCs, several studies have tested their antagonistic potential. For instance, the VOCs produced by endophytic bacteria such as *Pseudomonas* sp. Ba35, *Pseudomonas* sp. Sn48, *Pseudomonas* sp. Ou22, *Pantoea* sp. Sa14, *Serratia* sp. Ba10, and *Enterobacter* sp. Ou80 significantly decreased crown gall symptoms and inhibited the growth of *Agrobacterium tumefaciens* Gh1 (Etminani et al., 2022). Similarly, *Bacillus velezensis* strains (BUZ-14, I3, and I5) were effective against *Botrytis cinerea*, *Monilinia fructicola*, *Monilinia laxa*, *Penicillium digitatum*, *P.* *italicum*, and *P. expansum* (Calvo et al., 2020), while VOCs produced by *Bacillus*, *Psychrobacillus*, *Peribacillus*, *Pseudomonas*, and *Staphylococcus* strains inhibited the growth of *Fusarium solani*, *Fusarium oxysporum*, *Alternaria alternata*, *M. fructicola*, *B. cinerea*, *Sclerotinia sclerotiorum*, and *M. laxa in vitro* and *in vivo* (Toral et al., 2021).

In contrast, calcineurin is a Ca^2+^-activated phosphatase that derives stress responses in fungi and other eukaryotes. Pathogenic fungi use the calcineurin pathway to survive and flourish inside the host. Thus, calcineurin controls the virulence of pathogenic fungi. In natural environments, calcineurin is also important for cell vitality. By regulation of transcription factor Crz1p, several important physiological roles of calcineurin in RS are mediated (Malik et al., 2019). Due to its importance in fungal pathogenesis, calcineurin has received attention as a unique target of antimycotic therapy in different fungi such as *Cryptococcus* *neoformans*, *Candida albicans*, and *Aspergillus fumigatus* (Rose, 1971; Malik et al., 2019). However, little is currently known about the pathway of calcineurin in RS. Scientific knowledge on pathogen mechanisms and other factors of pathogen–host association is minimal, hampering the advancement of resistant genotypes. The transcription factor CRZ1 is a calcineurin downstream effector and is essential in the resistance of azole in *C. albicans* (Rose, 1971). However, a CRZ1 homolog in RS is still to be identified. The research of bioactive substances as CRZ1 protein inhibitors in RS is, therefore, of concern. Similarly, the S9 ribosomal protein is a crucial protein located in the ribosome in the mRNA entry channel. It plays an appropriate role in the initiation process of protein synthesis. It has been reported that protein S9 is essential for optimal cell growth and expansion, as S9 degradation has resulted in decreased protein synthesis associated with G1 cytotoxicity (Lindström and Zhang, 2008). Yet, more work on a specific antimycotic agent that targets the inhibition of the translation process during protein synthesis, which ultimately affects its function, will be of considerable importance. So, three-dimensional (3D) structures of CRZ1 and S9 protein were taken as the receptor for this study.

In this study, we aimed to explore the biocontrol activity of VOCs of PGPR against fungal phytopathogen RS. Biochemical and physiological responses of BLSB-infected maize plants exposed to VOCs were also evaluated. Furthermore, we assessed the antagonistic mechanism of VOCs by investigating the binding ability of the VOCs with suitable RS receptors such as CRZ1 and S9 protein by *in silico* methods.

## Materials and methods

### Bacterial strains

Fifteen rhizobacterial strains having plant growth-promoting traits were obtained from the Applied Microbiology and Biotechnology Lab (AMBL), Department of Biosciences, COMSATS University Islamabad (CUI), Pakistan. The characteristics and the ability of the strains to alleviate different stresses in various crops have been documented by Yasmin et al. (2021). For the revival, the strains were cultured on Luria-Bertani (LB) medium plates at 37°C for 24 h (Quiroz-villareal et al., 2012).

### Antagonistic activity of volatile organic compounds produced by plant growth-promoting bacteria

The antagonism of various compounds released by the PGPR against RS was evaluated by plate assays. The stock culture of RS was accessed from the AMBL, CUI, Pakistan. Potato dextrose agar (PDA) medium plates were used for the revival of RS. The plates were inoculated with a mycelium plug, sealed, and kept at 28°C ± 2°C for 7 days to achieve complete fungal growth. Pure cultures thus obtained were used for further analysis. To access the antagonism of VOCs produced by PGPR strains, the dual culture technique was used with minor modifications. Each bacterial strain (1 × 10^6^ CFU) was co-inoculated with a 4-mm plug of RS mycelium on the same Petri plate. The Petri plates were kept at an incubation temperature of 30°C in the dark, and mycelial growth was recorded after 6 days (Hasan, 2018). The positive control for this assay included PDA plates inoculated with only RS. The percentage inhibition was computed as follows:


Inhibition percentage=C−T/ C×100

where

C = pathogen radial growth in the positive control, T = pathogen radial growth in the dual culture plate

### Pot experiment

To evaluate the potential of bacterial VOCs to boost plant growth and reduce BLSB disease incidence in maize, a pot experiment was conducted at CUI, Pakistan, greenhouse (33.7294°N, 73.0931°E, with 15°C average temperature and 64% humidity). The experiment was laid out as a completely randomized design (CRD) with three replications for each treatment and five plants per pot during the maize-growing season, i.e., March 2022.

### Exposure of maize seeds with volatile organic compounds released by plant growth-promoting rhizobacteria

Seeds of maize variety “Soan 3” were obtained from the National Agriculture Research Centre (NARC), Islamabad, Pakistan. For surface sterilization, seeds were soaked in 70% ethanol followed by soaking in 5% NaClO solution for 2 min each and ultimate washing with sterilized distilled water (Tayyab et al., 2020). Bacterial cultures grown overnight in Murashige and Skoog (MS) media (1 × 10^6^ CFU) were used. The bacterial cultures were kept separated from maize seeds to avoid physical contact using two Petri plates. Maize seeds were grown in one Petri plate, while the PGPR strains were inoculated in another MS medium plate. The separate Petri plates with planted seeds and PGPR strains were incubated for 48 h at 30°C in a closed container (airtight plastic box). Seeds were watered with sterile deionized water. For seed germination, the boxes were kept in the dark and were later on shifted to the growth room with light and dark periods of 13/11 h for 10 days. The control seeds were incubated alone without exposing them to the bacterial VOCs.

Seedlings exposed to VOCs and unexposed (control) seedlings of identical size were selected and sown in pots (15 cm length × 13 cm width) filled with 1.5 kg sterilized soil and clay (3:1). Four treatments (T1 = Control without inoculation, T2 = RS, T3 = VOCs-exposed seedling, T4 = VOCs-exposed seedling infected with RS) were included in the experimental design.

Optimum moisture level and submerged conditions as per the requirement of maize plant were maintained by watering. Maize BLSB causal agent RS were grown for 6–7 days on PDA plates at 28°C ± 2°C. A 0.5% gelatin solution was used to harvest fungal spores, and a spore density of 10^5^ mL^-1^ was obtained by arranging the spores. The spore suspension (25 mL) was sprayed to maize plants. Black polyethene bags were used to cover the pots for 24 h to arouse the infection. The plants were infected with RS after 3 weeks of germination. The plants were harvested after 7 days of infection when symptoms of BLSB diseases were noticeable. Phenotypic traits like root and shoot lengths and biomass were then recorded, and root and shoot samples were preserved for postharvest biochemical assays.

### Relative water content

The fresh weight (FW) of leaves from each treatment was measured. These leaves were then dipped in distilled water for 24 h. After that, a complete turgid weight (TW) of leaves was taken. The leaves were then dehydrated in an oven for 72 h at 72°C until constant weight was obtained to get the dry weight (DW). The relative water content (RWC) was calculated using the following formula (Weatherley, 1950):


$$\text{RWC }\left(\%\right)=\left[\frac{\text{FW}-\text{DW}}{\text{TW }-\text{DW}}\right]\times \;100$$

### Photosynthetic pigments

The chopped leaf sample (0.05 g) was dipped into dimethyl sulfoxide (10 mL) and incubated for 4 h at 65°C in a water bath. The absorbance of the supernatant was then recorded at 663, 645, and 480 nm using the spectrophotometer (Specord® 200 PLUS, Germany) for chlorophyll *a*, chlorophyll *b*, and carotenoids, respectively (Shoaf and Lium, 1976). The following formulae were used to calculate the values of photosynthetic pigments:


$$\text{Chloropyll}\;\text{a }\left(\text{mg}/\text{g}\right)=\;\left[12.7\left(\mathrm{OD}\;663\text{nm}\right)-\;2.69\;\left(\mathrm{OD}\;645\text{nm}\right)\right]\times \frac{V}{1000}\times \;W$$

$$\text{Chlorophyll b }\left(\text{mg}/\text{g}\right)=\;\left[22.9\left(\text{OD }645\text{nm}\right)\;–\;4.68\;\left(\text{OD }663\text{nm}\right)\right]\times \frac{\text{V}}{1000}\times \text{ W}$$

Carotenoids (mg/g) = [OD 480nm + 0.114 (OD 663nm) – 0.638 (OD 645nm)

where

V = volume of extract

W = weight of sample

### Total soluble protein

Fresh leaves (0.1 g) were crushed in phosphate buffer (1 mL, pH = 7). The crushed leaves were centrifuged for 10 min at 3,000 rpm. Afterward, an equal volume of alkaline CuSO_4_ (Fehling Solution) was added to 0.1 mL of the supernatant and mixed for 10 min. Then, Folin reagent (0.1 mL) was added to the solution, and the final solution was incubated at 28 ± 2°C for 30 min. The absorbance of each sample and the blank solution (phosphate buffer, pH = 7.5) was recorded at 650 nm. The bovine serum albumin (BSA) calibration graph was used as a standard to quantify the protein (Lowry et al., 1951).


Total soluble protein (µg/g FW)= (C×V)/ Vt×W)

where

C = soluble protein in enzyme sample used
V = volume of the buffer solution
Vt = volume of enzyme extract used for reaction
W = fresh weight (0.5 g) of sample taken

### Proline

For quantitative analysis of proline, 0.1 g of foliage was crushed in 80% ethanol and incubated at 80°C in a water bath for 1 h. Following centrifugation at 12,000 rpm, the supernatant was filtered for 10 min. After that, 1 mL of 5% phenol and 0.5 mL of distilled water were added to the supernatant. The supernatant was incubated for 1 h, sulfuric acid (2.5 mL) was added, and then the absorbance was recorded at 490 nm (Bates et al., 1973). Proline content was computed as follows:


Proline (µg/g FW)= (C×V)/ (a×W)

where

C = proline content calculated from the standard curve
V = volume of solution
a = volume of supernatant
W = sample weight

### Malondialdehyde

Lipid peroxidation was assessed by estimating the malondialdehyde (MDA). To achieve a final volume of 8 mL, 0.5 g of leaf and root samples were mashed and homogenized with phosphate buffer (100 mM, pH = 8). Centrifugation of the samples was carried out for 15 min at 12,000 rpm at 4°C. Five percent of trichloroacetic acid (TCA) and thiobarbituric acid (TBA) constituted the reaction mixture. The enzyme extract (1.5 mL) and the reaction mixture (2.5 mL) were heated in a water bath at 95°C for 15 min and were then centrifuged for 10 min at 4,800 rpm to collect the supernatant. The absorbance was noted at 532 nm by a spectrophotometer. The blank reaction mixture contained TCA and TBA except for plant tissue (Buege and Aust, 1978).


MDA (nmol/gFW)= [(OD 532−OD 600)×A ×V)]/(a×E×W)

where

A = total reaction system + enzyme extract
V = total volume of buffer to extract enzyme
a = volume of enzyme extract
E = activity constant value, i.e., 0.155 mM/cm
W = fresh weight of sample

### Electrolyte leakage

The discharge of electrolytic ions from leaves was assessed to determine membrane electrolytic leakage (Rai et al., 2013). Initial water conductivity (initial cond.) was recorded immediately with the help of electrical conductivity (EC) detector after dipping 0.2 g leaf sample (without midrib) in deionized water. The EC was measured again after incubating the samples for 3 h at 25°C in a shaking water bath (Cole-Parmer, Illinois, USA) (cond. 3 h). Complete EC (max. cond.) was accessed by boiling the mixture in a water bath for 5 min. The following formula was used to determine the electrolytic leakage:


 [(cond. 3 h – initial cond) / (max.cond.–initial cond.)] ×100

### Total soluble sugars

The foliage extract was prepared by crushing 0.5 g of leaves with 1 mL of deionized water. The extract was then centrifuged for 10 min at 4,000 rpm. The supernatant was added with 0.3 mL phenol (5%) and 1 mL H_2_SO_4_ (98%), followed by incubation for 60 min at room temperature. The absorption spectrum was then observed at 485 nm using a spectrophotometer (DuBois et al., 2002).


TSS (mg/g)= OD ×K value× dilution factor/weight of sample×100

where

K value = 0.08

Dilution factor = 1^-1^

### Free amino acids

To determine the free amino acid content, pyridine (10%), ninhydrin solution (2%), and the leaf extract comprised the reaction mixture. The reaction mixture was boiled in a water bath for 30 min, and the optical density was recorded using a spectrophotometer at 570 nm (Hamilton and Van Slyke, 1943).

### ROS-scavenging enzyme analysis

#### Formulation of enzyme extracts

The 0.5 g of each of the root and shoot samples were crushed separately in a 5 mL phosphate buffer (100 mM, pH 7.8) for sample preparation. The crushed samples were centrifuged at 5,000 rpm for 20 min using SIGMA 3-18K Centrifuge (Germany). To prevent enzyme breakdown of the extract, the supernatant was preserved away from the light at 4°C.

#### Superoxide dismutase activity

A measure of superoxide dismutase (SOD) enzymatic activity is the frequency of inhibition in the photoreduction of nitro blue tetrazolium (NBT). A blend of 75 µM NBT, 20 µM riboflavin, 100 µM EDTA, 130 µM methionine, 0.25 mL distilled water, and 0.025 mL enzyme extract constituted the reaction mixture for SOD estimation (Beyer and Fridovich, 1987). To catalyze the reaction, the mixture was kept under 15 W fluorescent lamps at 4,000 lx for 10 min. Variation in the absorbance caused by the degradation of NBT was quantified via spectrophotometer at 560 nm and compared with blank (mixture without enzyme extract). The SOD activity was shown as U/g FW (fresh weight of shoot/root sample). In this study, 1 U (unit) is expressed as the enzyme initiating 50% inhibition of NBT. The SOD activity was determined by the formula:


SOD (U/g FW)=(Ack−Ae) ×V/ (0.5×Ack×W×Vt)

where

Ack = OD of light control
Ae = OD of sample
V = total volume of buffer used to extract enzyme
W = fresh weight of sample

#### Ascorbate peroxidase activity

To estimate the ascorbate peroxidase (APX), 0.1 mL each of enzyme extract, ascorbate peroxide (7.5 mM), H_2_O_2_ (300 mM) and 2.7 mL of potassium phosphate buffer (50 mM) were used as reagents. The standard reaction mixture formulated for zero reading included distilled water instead of the enzyme extract. Absorption was obtained at 290 nm in the time range of 0–60 s (Starlin and Gopalakrishnan, 2013).

#### Catalase activity

A few alterations were made in the method of Mendoza et al. (2018) to assess the catalase (CAT) activity. A blend of extracted enzyme solution (0.1 mL), H_2_O_2_ (0.1 mL, 300 mM), and phosphate buffer (2.8 mL, 50 mM) constituted the reaction mixture. The absorbance was estimated at 0 and 3 min at 240 nm using a spectrophotometer.

#### Peroxidase activity

The reaction mixture to assess the activity of peroxidase (POD) contained 1% H_2_O_2_, enzyme extract, and 0.05 M pyrogallol at a 1:1:3 ratio. The blank mixture content was the same, except that 0.5 mL of 0.1 M phosphate buffer (pH = 7) was used in place of the enzyme extract. The reaction mixture was incubated at 28 ± 2°C for 10 min. Then, at intervals of 30 s, 1 min, and 3 min, the absorption spectrum of the reaction mixture was observed at 240 nm via spectrophotometer (Vetter et al., 1958). After taking the absorption at relative wavelengths, APX, CAT, and POD of the samples were calculated using the following formula:


APX/CAT/POD (mM/g FW)=(OD ×A×V/A)/ (E×W)

where

A = amount of enzyme used in mixture
V = total volume of buffer used to extract enzyme
E = activity constant value (APX = 2.8 mM/cm, CAT = 39.44 mM/cm, POD = 22.9 mM/cm)
W = fresh weight of sample

#### Polyphenol oxidase activity

The 1.5 mL sodium phosphate buffer (0.1 M, pH = 6.5) and 200 μL catechol (0.01 M) were added to the enzyme extract (200 μL) to compose the reaction solution. In the blank solution, the enzyme extract was substituted with distilled water. The absorbance was recorded at 496 nm at intervals of 30 s for 3 min. The polyphenol oxidase (PPO) activity was measured at 485 nm/min/mg of protein as a difference in absorbance (Kahn, 1975).


PPO (mMgFW)=(OD at 3 min−OD at 0 min×total reaction vol)(time interval×0.2 )

#### Phenylalanine ammonia lyase activity

The reaction solution to examine the phenylalanine ammonia lyase (PAL) activity consisted of enzyme extract (30 μL), 670 μL of 3 mM L-phenylalanine, and 300 μL of distilled water. The composition of the blank mixture was same, except that the enzyme extract was replaced with 150 mM of Tris HCl buffer (pH 8.5). At 30 s intervals, the absorbance was read spectrophotometrically for 3 min at 270 nm (Suzuki et al., 2003).


PAL (U/ml)=(OD 270−OD 270 blank)(V×Dilution factor)/ (19.73×0.1)

where

V = total volume of the reaction mixture

19.73 = millimolar extinction coefficient of transcinnamate

Dilution factor = 1^-1^

#### Phytohormone analysis

The concentrations of phytohormones, i.e., indole acetic acid (IAA), gibberellic acid (GA), and abscisic acid (ABA), in maize foliage were estimated by extracting the hormones in 80% methanol (Kettner and Dörffling, 1995). The solvent collected was retained and substituted successively for 3 days with an equivalent amount of fresh methanol. The volume of the filtrate was reduced to half using a thin-film rotary evaporator (Cole-Parmer, Illinois, USA) at 35°C after centrifugation at 10,000 rpm.

For partitioning of the concentrate, half the volume of ethyl acetate (pH 2.3–3.0) was used four times. The ethyl acetate collected was then dehydrated using a thin-film rotary evaporator and dissolved in 1 mL of 100% methanol. Phytohormones of the sample were analyzed using high-performance liquid chromatography (HPLC) (Agilent 1100, Germany). The mobile phase consisted of methanol, acetic acid, and water (30:1:70).

### *In silico* analysis

*In silico* analysis was carried out to investigate the binding affinity of the identified VOCs (ligands) with suitable RS receptors.

#### Ligand preparation

The 3D chemical structures of ligands 2-pentylfuran, 2,3-butanediol, and dimethyl disulfide were obtained from PubChem (https://pubchem.ncbi.nlm.nih.go) in SDF format. Before docking, the SDF format was converted to PDB using an online converter (https://cactus.nci.nih.gov/translate/).

#### Receptor preparation and estimation of binding pockets

Among the RS targets, CRZ1 (Malik et al., 2019) and S9 protein (Islam et al., 2020) were selected as receptors based on their important physiological functions. The structures of the selected receptors (CRZ1 and S9 protein) were not present in the Protein Data Bank (PDB) (http://www.rcsb.org). Thus, the sequences of amino acids for CRZ1 and S9 protein were recovered from the NCBI database (http://ncbi.nlm.nih.gov/) using accession numbers QNH90388.1 and ABE68880, respectively. Then, the SWISS model was used to predict the structure of receptors (CRZ1 and S9) in RS, but the parameters used to show its quality like identity and local quality estimates and comparison with a nonredundant set of PDB structures showed the low quality of the predicted models by this method. So, we used I-TASSER to predict the receptor homology models. After getting the five models based on 10 templates, active binding site residues for CRZ1 and S9 receptors were predicted by I-TASSER using meta server approach of COFACTOR and COACH (**Table 1**).

**Table 1:** List of active ligand-binding site residues of CRZ1 receptor in maize banded leaf and sheath blight (BLSB) pathogen *Rhizoctonia solani* (RS).

Binding Pockets	Ligand-Binding Site Residues in RS	
	CRZ1	S9
**Binding Pocket 1**	665, 670, 683, 687	6, 7, 8, 9, 10, 11, 12, 14, 15, 16, 17, 18, 19, 21, 37, 38, 39, 40, 44, 50, 53, 54, 71, 72, 75, 82, 120, 121, 123, 124, 126, 127, 130, 131, 132,133, 140, 142, 143, 144, 145, 146, 149, 163, 168, 169, 170, 171, 172, 173
**Binding Pocket 2**	600, 635, 639, 641, 642, 643, 648, 671, 672, 675, 678, 679, 682, 714, 717, 721, 724	52, 53, 56, 73, 96, 97
**Binding Pocket 3**	440, 473, 481, 511, 514, 515, 518, 538	10, 11, 12, 14, 16, 17, 18, 19, 21, 24, 28, 29, 31, 32, 37, 38, 39, 40, 43, 44, 50, 53, 54, 55, 57, 72, 75, 79, 82, 120, 121, 123, 124, 126, 130, 131, 139, 140, 142 ,143, 144, 146, 16, 163
**Binding Pocket 4**	450, 457, 460, 463, 469, 479, 489, 491, 493, 496, 497, 500, 507	35, 37, 123
**Binding Pocket 5**	579, 584, 587, 589, 590, 595, 601, 602, 605, 608, 609, 613, 614, 617, 618, 621 ,629 ,633, 636	126, 144

#### Molecular docking

Virtual screening tool PyRX was used for docking analysis. The simulation between receptors (CRZ1 and S9) and ligands (2-pentylfuran, 2,3-butanediol, and dimethyl disulfide) was performed using Vina wizard. The energy of the uploaded ligands was minimized. **Table 2** shows the parameters used for Vina grid and spacing. To observe all of the possible confirmations, nine runs were set with optimum grid size to cover the entire receptor. Also, the docking result was analyzed between ligand and receptor binding energies and binding interactions. Parameters used in Vina search space for docking were center (Å), X: –27.5501, Y: –0.2162, and Z: 50.3675 and Dimensions (Å) included X: 65.5753, Y: 62.4444, and Z: 57.9704.

**Table 2:** Binding energy and active residues of CRZ1 and S9 protein receptor in blind docking of 2-pentylfuran, 2,3-butanediol, and dimethyl disulfide.

Receptors	Ligands	Active Residues	Binding Energy
**CRZ1**	**2-pentylfuran**	Lys595, Leu635, Ala648	-4.3
	**2,3-butanediol**	–	
	**Dimethyl disulfide**	–	
**S9**	**2-pentylfuran**	Ile12, Val14, Pro15, Trp43, Arg44, Ser50	-4.1
	**2,3-butanediol**	Val14, Pro15, Leu24, Ala31, Asn38, His123	-3.2
	**Dimethyl disulfide**	Thr11, Ile12, Val14, Pro15, Arg40, Trp43, Arg44, ser50, Arg82	-2

#### Post-docking analysis

For post-docking analysis, Chimera Ligplot+ and VigaZZ were used. Vigaz appended the docked molecules and showed nine frames for each run. Then, each frame was observed on Ligplot+ for binding site conformations. The docking analysis was carried out to compare the binding sites of the docked molecule to the actual active binding sites to check the success of docking.

### Statistical data analysis

One-way analysis of variance (ANOVA) was applied to statistically evaluate the data using Statistix software (version 8.1). Means of three replicates were compared using the least significant difference (LSD) test at *P* ≤ 0.05 (Steel and Torrie, 1962).

## Results

### Antagonistic activity of the volatile organic compounds against *Rhizoctonia solani*

Among 15 PGPR strains tested for antagonistic activity of their VOCs against RS, 10 strains showed antagonism (**Table 3**). The antagonistic potential of VOCs of the tested PGPR strains ranged from 50% to 80% as compared to the control (without PGPR). The maximum antagonistic activity (80%) was displayed by PGPR strain *P. pseudoalcaligenes* SRM-16 (Accession Number: MG736962).

**Table 3:** *Rhizoctonia solani* (RS) suppression by antagonistic bacteria.

Sr No.	Strain Name	Strain Code	Accession Number	% Inhibition of RS ± SE
**1**	*B. subtilis*	SRM-3	MG733991	0
**2**	*B. megaterium*	SRM-9	MG73398	0
**3**	*P. pseudoalcaligenes*	SRM-16	MG736962	80±1^a^
**4**	*Bacillus* sp.	SRM-20	MG733990	55±4.7
**5**	*B. megaterium*	MU2	MG562498	0
**6**	*B. licheniformis*	MU8	MG012485	66.9±1.4
**7**	*P. aeruginosa*	KFP1	HQ007938. 1	64.2±1.4
**8**	*P. aeruginosa*	KFP2	Q007939. 1	56.8±1.6
**9**	*P. aeruginosa*	KFP1	Q007940. 1	58.5±1.5
**10**	*P. putida*	CW1	MT604992	65.2±2
**11**	*Proteus* sp.	HY13KI	KJ191560.1	0
**12**	*Pseudomonas* sp.	HY13KR	KM016981.1	61.9±1.1
**13**	*B. pumilus*	HYJW	KT003271.1	64.2±1.1
**14**	*Pseudomonas sp.*	HY8N	KJ191560.1	58.5±1.5
**15**	*B. cereus*	HY9K	DQ289055.1	0

Mean values of three replicates ± standard error (SE) are presented, and the values bearing different letters are significantly different from each other based on Fisher’s least significant difference (LSD) test at P ≤ 0.05.

### Effect of *Pseudomonas pseudoalcaligenes* SRM-16 VOCs-exposed maize seedling on BLSB progression

During the pot experiment, VOCs released by *P. pseudoalcaligenes* SRM-16 helped reduce disease incidence and promoted the growth of plants indicated by both phenotypic and physiological changes.

### Banded leaf and sheath blight disease score

**Figure 1:**
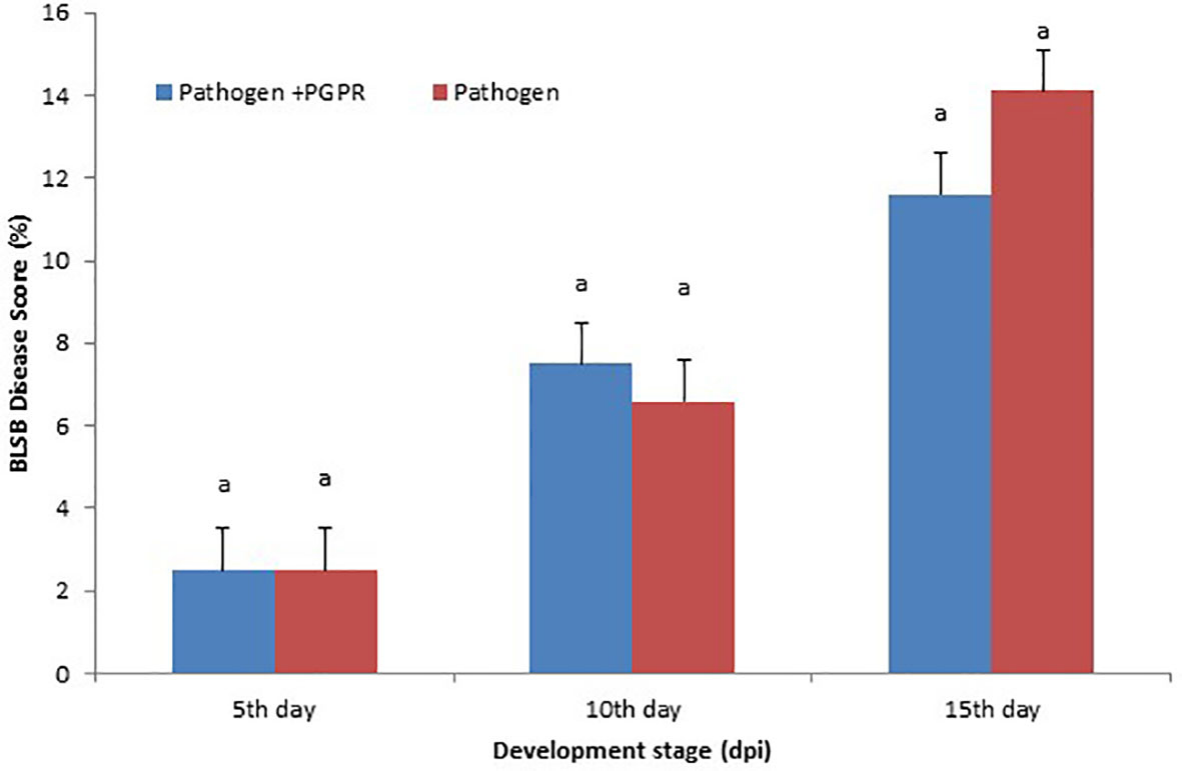
Area under the disease progress curve (AUDPC): Maize banded leaf and sheath blight (BLSB) disease score (15 days post-inoculation). Same letter above bars indicate statistically non-significant difference between treatments at P ≤ 0.05.

The disease was scored according to the area under the disease progress curve (AUDPC) scale, which gives the quantitative summary of results of disease intensity over a specific time period, so that the results can be easily compared to the control. The minimum disease score was recorded when seedlings were exposed to the VOCs compared to the BLSB-infected maize plants. The VOCs-exposed maize showed 11.6% disease incidence compared to the non-inoculated maize with 14.1% disease incidence (**Figure 1**).

### Plant growth and biomass

**Figure 2:**
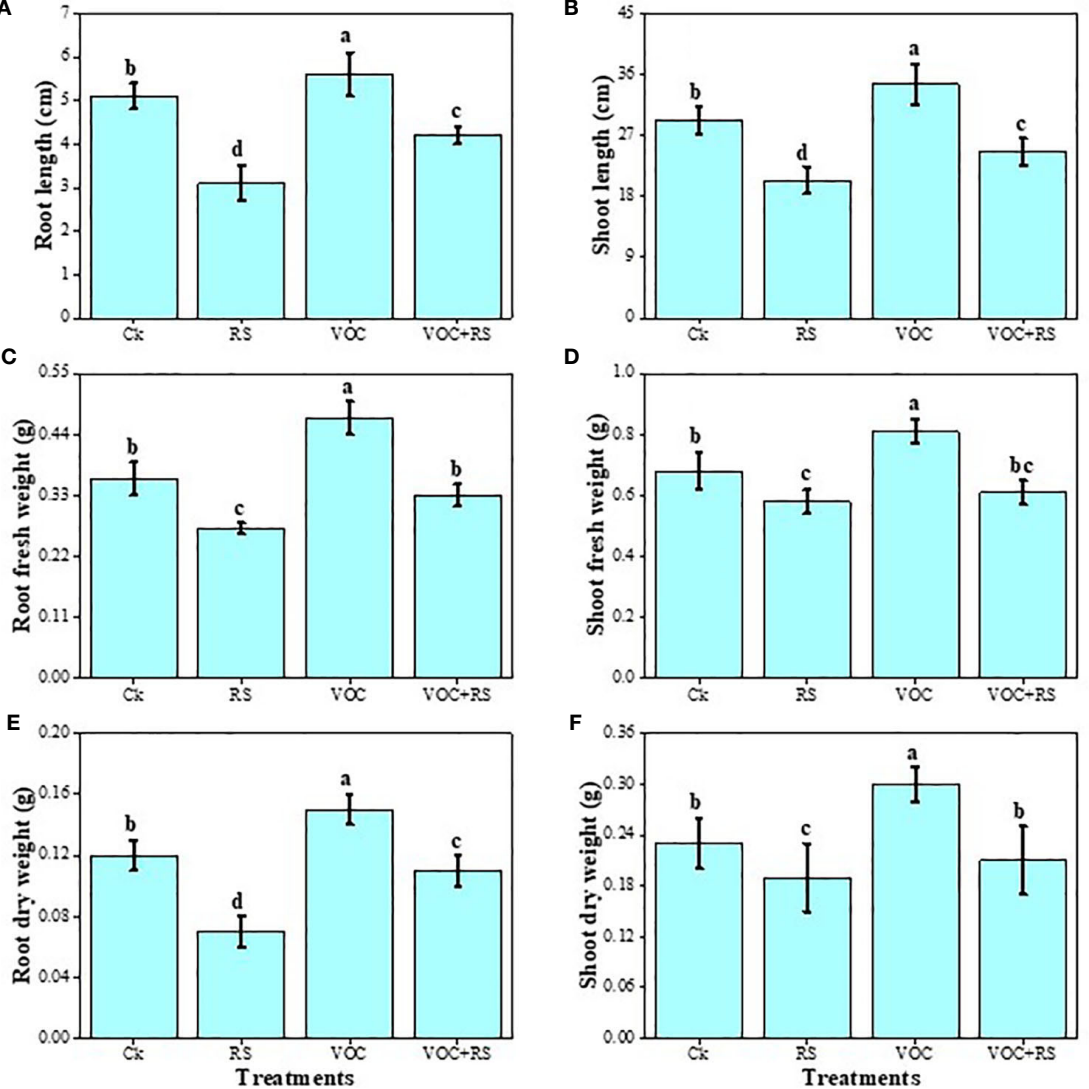
Effect of different treatments of RS and VOC on various growth parameters of maize, i.e., **(A)** root length, **(B)** shoot length, **(C)** root fresh weight, **(D)** root dry weight, **(E)** shoot fresh weight, and **(F)** shoot dry weight. Bars with different letter(s) for each parameter differ significantly at *P* ≤ 0.05. Mean values of three replicates are presented (n = 3). Error bars show standard deviation (SD). Ck (control), RS (*Rhizoctonia solani*), VOC (volatile organic compound), and VOC+RS (volatile organic compound along with *R. solani* infection). Different letters above bars indicate statistically significant difference between treatments at P ≤ 0.05.

The data regarding various growth and biomass-related attributes of maize are presented in **Figure 2**. Our results advocated that RS-infected plants showed a significant decrease in root length, shoot length, shoot fresh weight, shoot dry weight, root fresh weight, and root dry weight by 37.3%, 62.8%, 17.6%, 19%, 12.6%, and 24.6%, respectively, compared to those of the control. However, the application of VOCs increased all growth parameters individually or in combination with RS. Compared to the plants without RS treatment (control), the highest increase of 29.1%, 16.5%, 64.8%, 16.7%, 34.3%, and 13.4% were recorded in the root length, shoot length, shoot fresh weight, shoot dry weight, root fresh weight, and root dry weight of the plants grown in the presence of VOCs, respectively. A slight decrease in the growth and biomass of plants was observed under the combined treatment of VOC and RS.

### Gas exchange characteristics and photosynthetic pigments

**Figure 3:**
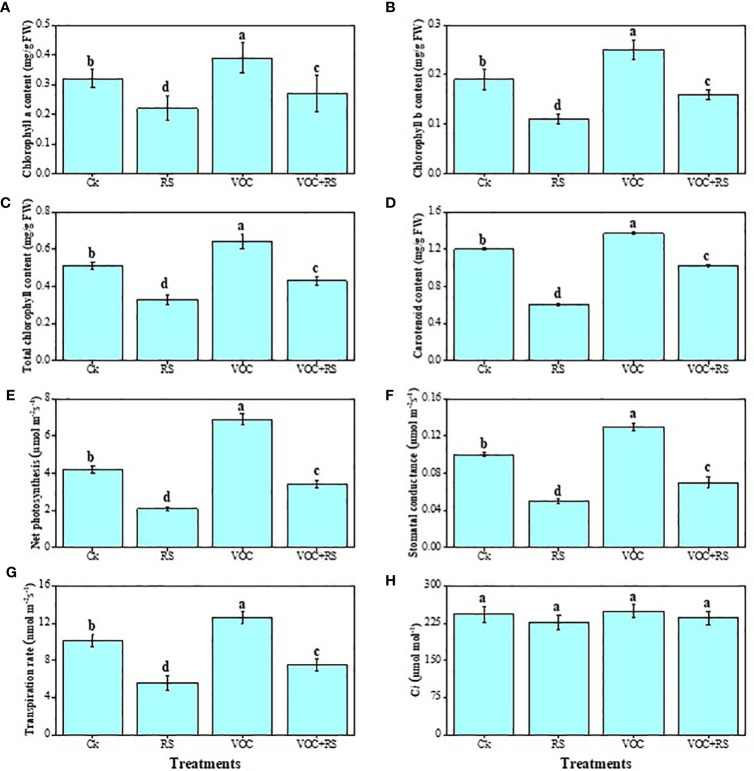
Effect of different treatments of RS and VOC on different photosynthetic measurements, i.e., **(A)** chlorophyll a content, **(B)** chlorophyll b content, **(C)** total chlorophyll content, **(D)** carotenoid content, **(E)** net photosynthesis, **(F)** stomatal conductance, **(G)** transpiration rate, and **(H)** intercellular CO_2_ on maize plants. Bars with same letter(s) are not significantly different at *P* ≤ 0.05. Data represent the mean (n = 3). Error bars indicate standard deviation (SD). The detail of treatments is the same as in **Figure 2**. Different letters above bars indicate statistically significant difference between treatments at P ≤ 0.05.

Except intercellular CO_2_ (C*i*), all other parameters dropped significantly under RS treatment, whereas treatment with VOCs significantly increased gas exchange characteristics and photosynthetic pigments (**Figure 3**). Compared to the control plants, total chlorophyll content, carotenoid content, net photosynthesis (P*_N_*), stomatal conductance (g*_s_*), and transpiration rate (*E*) were decreased by 29.4%, 67.7%, 46.3%, 87.6%, 69.8%, respectively, in the plants that were treated with RS. Although C*i* did not change significantly under any treatment of RS or VOCs, results, however, showed that VOC treatment significantly increased total chlorophyll and carotenoid contents, P*_N_*, g*_s_*, and *E* by 24.8%, 60.5%, 42.6%, 19.6%, and 67.7%, respectively, when compared to the control.

### Oxidative stress indicators and proline content

**Figure 4:**
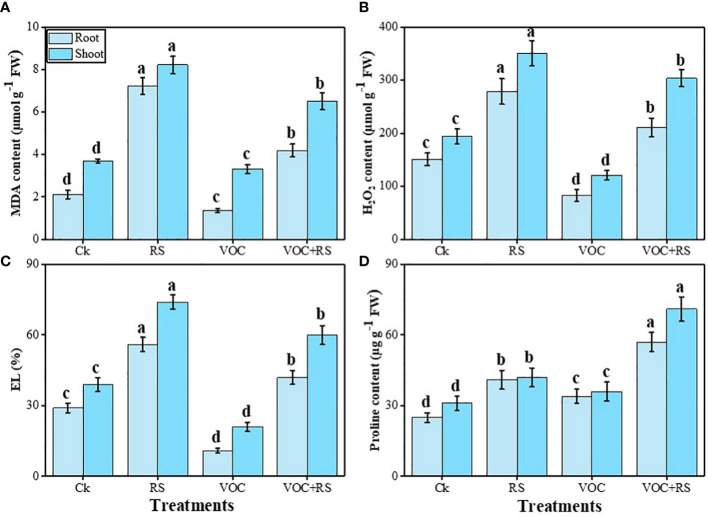
Effect of different treatments of RS and VOC on oxidative stress indicators, i.e., **(A)** malondialdehyde (MDA) content, **(B)** H_2_O_2_ initiation, **(C)** electrolyte leakage (EL), and **(D)** proline contents in the roots and shoots of *Zea mays*. Bars having different letter(s) are significantly different at *P* ≤ 0.05. Mean data are presented (n = 3). Standard deviation (SD) is represented by error bars. The detail of treatments is the same as in **Figure 2**. Different letters above bars indicate statistically significant difference between treatments at P ≤ 0.05.

The proline content was maximum in the plants grown in the presence of VOCs and RS compared to the control. In addition, proline contents and oxidative stress indicators were more pronounced in the shoots than those in the roots. Relative to the control, MDA content, H_2_O_2_ initiation, and EL were enhanced by 279.7%, 169%, and 151.9% in the roots and 316.6%, 179.9%, and 197.9% in the shoots, respectively, under RS treatment. Oxidative stress indicators were dropped in response to the combined application of VOCs and RS in both organs of the plants, which were further decreased by the individual application of VOCs. In contrast, proline content was increased by 141.3% in the roots and by 149.7% in the shoots compared to the plants grown under control conditions (**Figure 4**).

### Enzymatic-antioxidant capacity

**Figure 5:**
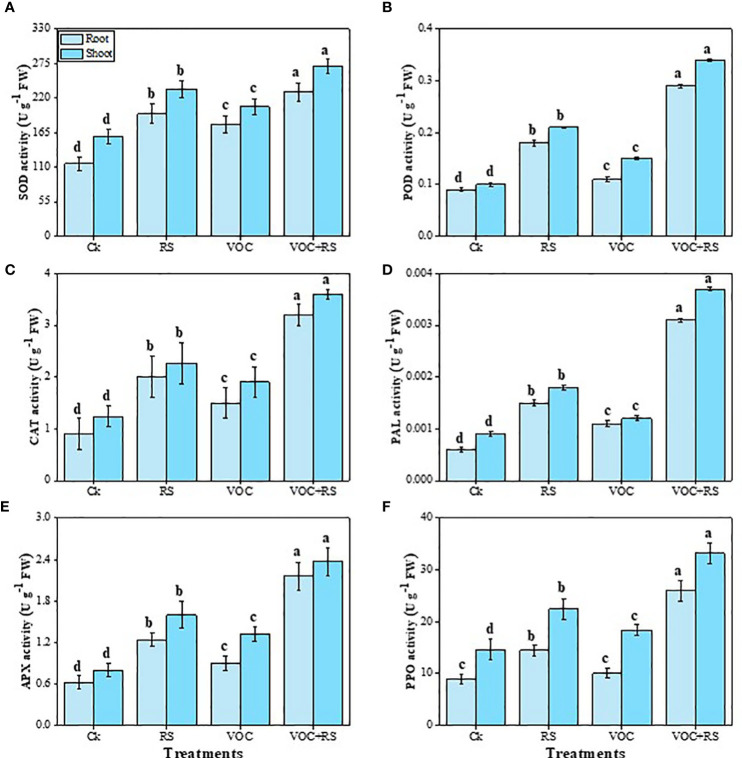
Effect of different treatments of RS and VOC on antioxidant capacity, i.e., **(A)** superoxidase dismutase (SOD), **(B)** peroxidase (POD), **(C)** catalase (CAT), **(D)** phenylalanine ammonia lyase (PAL), **(E)** ascorbate peroxidase (APX), and **(F)** polyphenol oxidase (PPO) activities of *Zea mays* roots and shoots. Bars sharing similar letter(s) are nonsignificantly different (*P* ≤ 0.05). Values are mean of three replications (n = 3). Standard deviation (SD) is represented by error bars. The detail of treatments is the same as shown in **Figure 2**. Different letters above bars indicate statistically significant difference between treatments at P ≤ 0.05.

The data indicated that the activities of SOD, POD, CAT, PAL, APX, and PPO were increased under the treatment of VOCs along with RS in a combined application (**Figure 4**). Our results depicted that the simultaneous application of RS and VOCs enhanced SOD, POD, CAT, PAL, APX, and PPO activities by 96.7%, 266.6%, 313.7%, 246.6%, 307%, and 149.7%, respectively, in the roots and by 81.6%, 246.4%, 269.5%, 269.6%, 329%, and 137.6%, respectively, in the shoots, relative to the control plants. However, the results obtained from the individual application of VOCs were less effective than the findings obtained from the individual application of RS in the roots and the shoots of *Zea mays* (**Figure 5**).

### Phytohormones, sugars, and amino acid content

**Figure 6:**
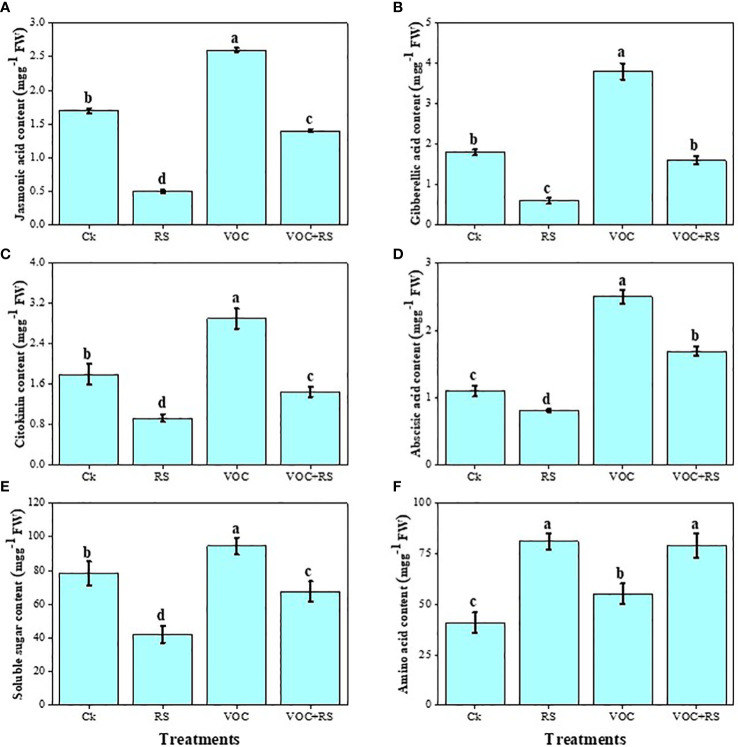
Effect of different treatments of RS and VOC on phytohormones and sugars, i.e., **(A)** jasmonic acid, **(B)** gibberellic acid, **(C)** cytokinin, **(D)** abscisic acid, **(E)** soluble sugars, and **(F)** amino acid content of *Zea mays*. Bars having different letter(s) for each parameter differ significantly at *P* ≤ 0.05. Values of three replicates are presented (n = 3). Standard deviation (SD) is indicated by error bars. The detail of treatments is the same as in **Figure 2**. Different letters above bars indicate statistically significant difference between treatments at P ≤ 0.05.

The findings showed that the level of different hormones and soluble sugar content declined under the application of RS while amino acid content boosted in the presence of RS compared to those of the control (**Figure 6**). Treatment with RS decreased jasmonic acid (JA), GA, cytokinin, ABA, and soluble sugar content by 73.9%, 19.7%, 27.6%, 7.4%, and 61.6%, respectively, compared to the plants without any application of RS and VOCs. In contrast, RS treatment increased amino acid contents by 43.9% when compared with those of the control. However, the highest contents of JA, GA, cytokinin, ABA, and soluble sugar were noticed under the treatment of VOCs, which were enhanced by 29.7%, 67.7%, 36%, 157.4%, and 19.2%, respectively, compared to those of the control.

### Relationship of the studied parameters with various treatments

**Figure 7:**
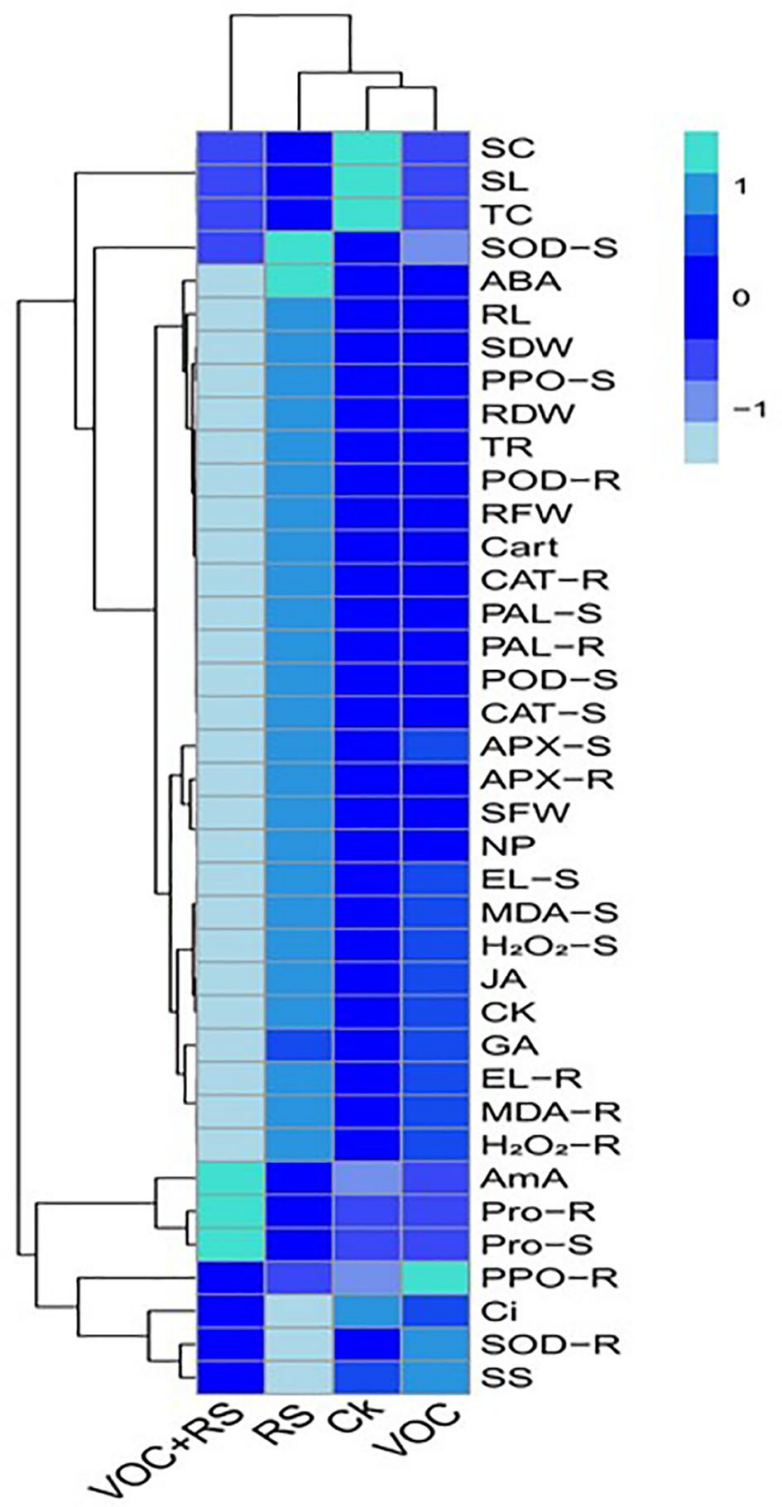
Heat-map histogram relationship between various studied parameters along with various incubation treatments of *Zea mays* plants. The detail of treatments is the same as in **Figure 2**. SC, stomatal conductance; SL, shoot length; TC, total chlorophyll content; SOD-S, shoot superoxidase activity; ABA, abscisic acid content; RL, root length; SDW, shoot dry weight; PPO-S, shoot polyphenol oxidase activity; RDW, root dry weight; TR, transpiration rate; POD-R, root peroxidase activity; RFW, root fresh weight; Cart, carotenoid content; CAT-R, root catalase activity; PAL-S, shoot phenylalanine ammonia lyase activity; PAL-R, root phenylalanine ammonia lyase activity; POD-S, shoot peroxidase activity; CAT-S, shoot catalase activity; APX-S, shoot ascorbate peroxidase activity; APX-R, root ascorbate peroxidase activity in the roots; SFW, shoot fresh weight; NP, net photosynthesis; EL-S, shoot electrolyte leakage; MDA-S, shoot malondialdehyde content; H_2_O_2_-S, shoot hydrogen peroxidase initiation; JA, jasmonic acid content; CK, cytokinin content; GA, gibberellic acid content; EL-R, root electrolyte leakage; MDA-R, root malondialdehyde content; H_2_O_2_-R, root hydrogen peroxidase initiation; AmA, amino acid content; Pro-R, root proline content; Pro-S, shoot proline content; PPO-R, root polyphenol oxidase activity; Ci, intercellular CO_2_; SOD-R, root superoxidase activity; SS, total soluble sugar content.

A heat-map histogram relationship was illustrated to depict a connection between the studied parameters and various treatments of incubation (**Figure 7**). Combinatorial treatment with VOCs along with RS showed a negative relationship with most of the growth parameters, antioxidant capacity, oxidative stress indicators, and phytohormones. The remaining parameters showed nonsignificant results with the rest of the treatments.

### Post-docking analysis

Molecular dockings were performed between CRZ1 and S9 receptor and VOCs 2-pentylfuran, 2,3-butanediol, and dimethyl disulfide using Autovina Pyrx plug in. The 3D structures of the ligands are shown in **Supplementary Figure S1**. Based on the energy values, the docking conformations were executed; thus, the first structure corresponds to the lowest energy conformation (**Supplementary Table S1**). The stability of a complex is related directly to the complex’s free energy. The presence of a high binding affinity between the ligand and the receptor indicates lower energy values. It is of great significance to illustrate the free energy within each framework to determine the effective strong interaction. On Ligplot, every complex was then analyzed to identify the receptor’s binding residues. Detailed examination of the conformations of the binding site showed that the 2-pentylfuran ligand binds to the CRZ1 protein receptor at active binding pockets. **Table 2** describes the overall binding trend of each conformation for VOCs 2-pentylfuran, 2,3-butanediol, and dimethyl disulfide.

**Figure 8:**
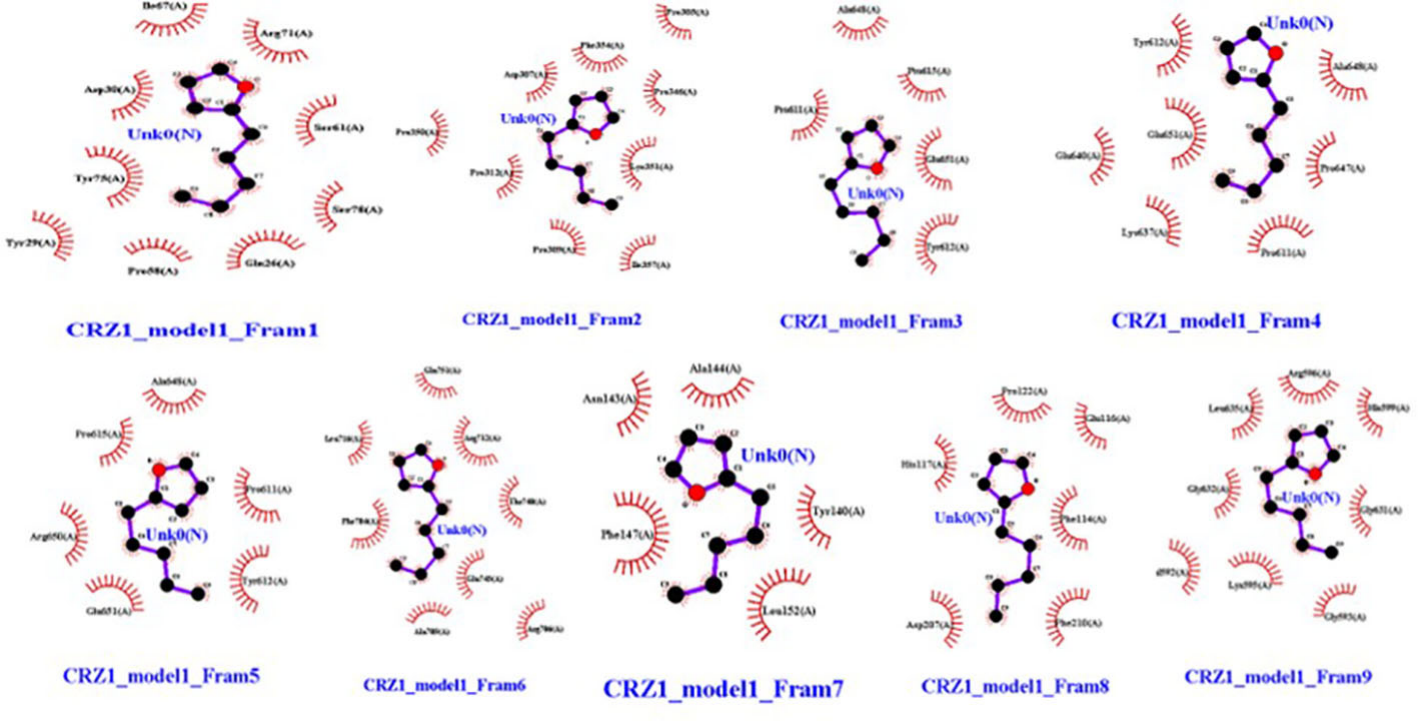
Interaction of bacterial VOC ligand 2-pentylfuran with CRZ1 receptors of *Rhizoctonia solani* in nine frames. Hydrophobic interactions are indicated by red residues, while hydrogen bond interactions are shown by atomic residues.

In frames 3, 4, and 5, we found 2-pentylfuran ligand attachment at Ala648 residue, while in frame 9, we found ligand attachment at Leu635 and Gly595 residues. The position of 2-pentylfuran is shown to bind to the same binding pockets as suggested by I-TASSER. **Figure 8** represents the graphical interactions. However, 2,3-butanediol and dimethyl disulfide were unable to form binding at any of the active binding pockets of receptor CRZ1 in all nine frames.

**Figure 9:**
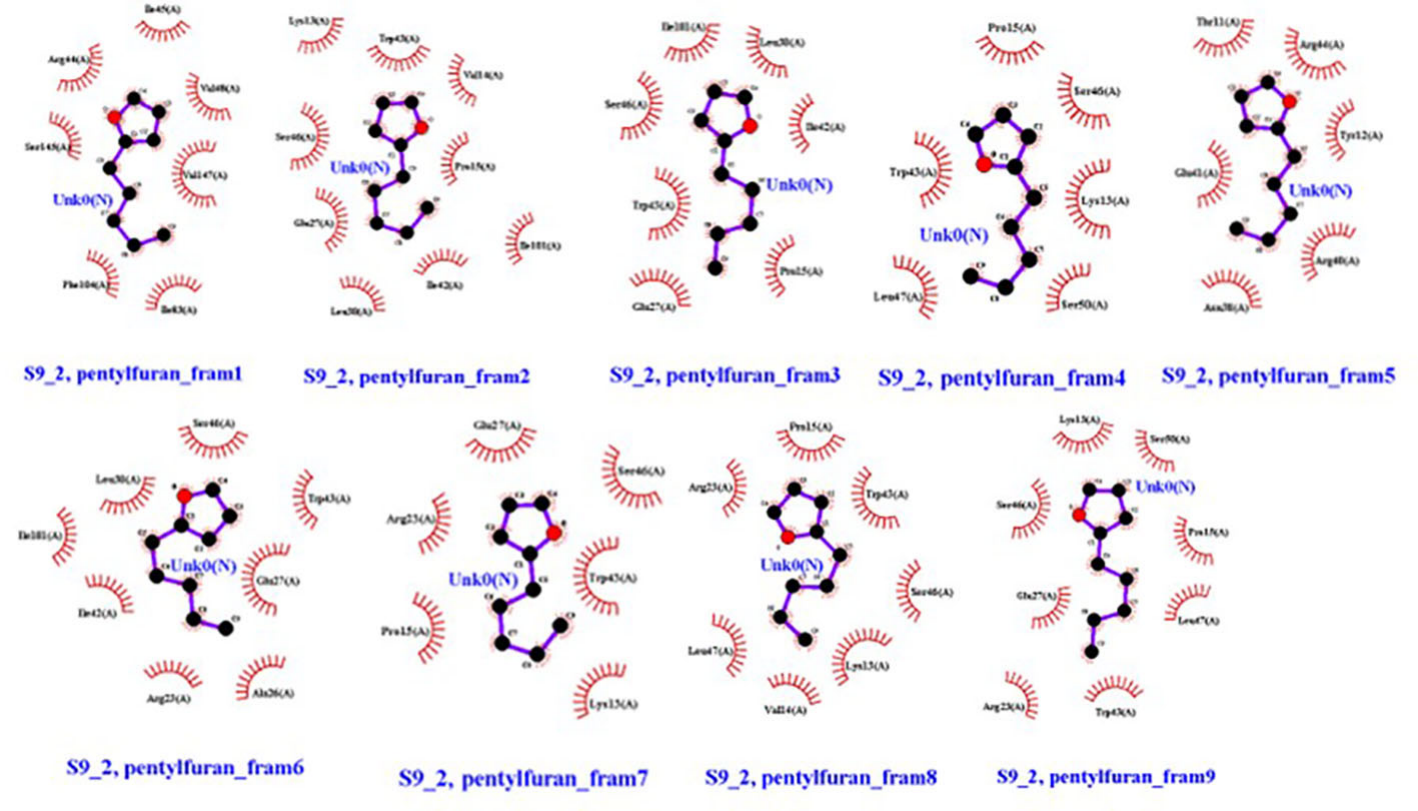
Ligands (VOC) 2-pentylfuran, interaction with S9 protein receptor of *Rhizoctonia solani* in nine frames. Hydrophobic interactions are indicated by red residues, while hydrogen bond interactions are shown by atomic residues.

Overall, 2-pentylfuran showed binding affinity with both receptors of RS, while 2,3-butanediol and dimethyl disulfide exhibited binding affinities with only S9 protein. Hence, it can be predicted that S9 protein receptors are more likely the targeted RS receptors of VOCs to inhibit the growth of RS (**Figures 8**, **9**).

## Discussion

PGPR positively affect both the physiological and phenotypical aspects of plant under biotic and abiotic stress conditions (Steel and Torrie, 1962; Chenniappan et al., 2019; Amna et al., 2020; Oleńska et al., 2020). Application of PGPR to antagonize the phytopathogen RS causing BLSB in maize is well established (Singh et al., 2020). Commonly studied biocontrol mechanisms used by rhizobacteria include secretion of hydrolytic enzymes, antibiotics, and bacteriocins (Khan et al., 2020; Riaz et al., 2021). However, the information about the target protein receptors of RS and their interaction with the active VOCs produced by PGPR is still insufficient.

Rhizobacteria produce several different VOCs that help in establishing the eco-friendly mechanism for promoting plant growth and stress alleviation without having physical contact with plants (Tilocca et al., 2020). In the present study, VOCs produced by *P. pseudoalcaligenes* SRM-16 exhibited the potential to alleviate the BLSB diseases in maize. Among 15 PGPR strains tested, the maximum antagonistic activity (80%) was shown by VOCs produced by *P. pseudoalcaligenes* SRM-1. Similarly, Agisha et al. (2019), Amna et al. (2020), and Yasmin et al. (2020b) have reported the antagonism of various PGPR strains against RS in different crops. It can be speculated that these VOCs can be formulated as antifungal compounds for disease suppression in crops.

The current study showed improved disease resistance of infected maize plants exposed to VOCs by altering morpho-physio-biochemical traits. In addition, VOCs inhibit the expression of virulence genes and also reduce the expression of antioxidant activity-related genes as well as the regulators that control virulence factors such as cell wall-degrading enzymes, extracellular polysaccharides, and bacterial motility. Although the inhibition of phytopathogen growth by bacterial VOCs is well known, the mechanism by which this inhibition is achieved is poorly understood. Current studies suggest that VOCs inhibit pathogens by hindering specific enzyme activity and affecting protein production and mobility that ultimately influence the proliferation, cell morphology, and virulence of phytopathogens. For example, pathogenic bacterial colonies are distorted and cells are damaged in the presence of VOCs. Moreover, VOCs supress bacterial biofilm production, motility, and antioxidant and exopolysaccharide production. In case of fungi, hyphal morphology is changed on exposure to VOCs. Moreover, VOCs can cause the enlargement of vacuoles in hyphae, mycelia, and conidiophores. This vacuolization can affect the cellular respiration of fungal pathogens, rupture hyphae, aggregate cytoplasm and protoplasm, degrade cell wall, and cause cell breakage and leakage of intracellular substances (Almeida et al., 2023).

Our results also indicated a pronounced stimulation in the development of plants exposed to VOCs produced by PGPR. A positive outcome of VOCs-exposed maize seedling under BLSB infection in a pot experiment was observed. Overall, maize seedlings exposed to VOCs showed a significant increase in disease tolerance by enhancing the biomass (root and shoot length and their fresh and dry weight) and the activity of plant antioxidant and defense enzymes as compared to the healthy and non-exposed seedlings. This could be attributed to reduced proliferation of pathogen in plants depicted by reduced disease incidence in treated plants than non-treated plants. Earlier studies have also demonstrated the beneficial role of endophytic bacteria in reducing disease incidence in maize (Omara et al., 2017; Singh et al., 2020). However, the role of VOCs as a biocontrol agent against RS is not reported yet. Moreover, improved biomass, reduced membrane lipid peroxidation, and higher proline and protein accumulation were also observed in infected roots and shoots under VOC treatment. However, a higher increase was observed in shoots as compared to roots. Since VOCs are volatile, they can diffuse from their application points to longer distances, allowing them to exert their inhibitory effects on the targeted pathogens without the need of direct physical contact between the target pathogen and the VOC-producing microorganism (Tilocca et al., 2020). This can be attributed to the induced systemic resistance of maize against RS infection (Bukhat et al., 2020). Other researchers have found *Bacillus amyloliquefaciens* to exhibit strong biocontrol activity against tomato pathogen *Ralstonia solanacearum*, but interestingly, there was no inhibition of the bacterial VOCs by using non-VOC-producing bacteria (Raza et al., 2016).

Infection-induced production of ROS is a known phenomenon in any organism. However, if they are exceeded beyond an optimum level, they cause damaging and toxic effects on cellular machinery. These damaging effects disturb photosynthetic machinery, soluble sugar content, total protein content, osmoregulatory mechanism, and cell membrane integrity. Defense systems of plants cope up with this stress by upregulating the detoxifying ROS scavenging enzymatic system. Our results revealed an enhanced release of defense enzymes such as SOD, POD, CAT, APX, PAL, and PPO in RS-challenged roots and shoots of maize; however, VOCs played a prominent protective role by further increasing their production. Antagonistic activities of 2,3-butanediol against *Enterobacter aerogenes*, a pathogen of maize plant, have already been observed (D'Alessandro et al., 2014).

In addition to the release of Reactive oxygen species (ROS) scavenging enzyme system under stress conditions, plant growth, development, and defense depend upon the balance and crosstalk between different phytohormones. Several studies have reported the essential role of JA, cytokinin, ABA, and GA in inducing systemic resistance (ISR) under pathogen infection. However, during ISR, signal transduction majorly depends upon the JA- and minorly upon the salicylic acid (SA)-mediated pathway. It is known that beneficial microorganisms activate both the pathways to minimize the infection caused by plant pathogens. However, in this study, we report for the first time an ameliorative potential of VOCs by enhancing the production of stress hormones in infected maize plants (**Figure 7**). Hence, several physiological and biochemical mechanisms worked together to induce VOC-mediated disease resistance in maize. However, a detailed insight is needed to understand the signal transduction and crosstalk between different factors to understand the non-physical impact of VOCs to counteract the devastating effects of RS.

Understanding the molecular mechanisms of antagonism through which VOCs reduce disease incidence in plants is of great significance (Islam et al., 2020). Protein ligand interaction has already been reported in many studies that help to understand the mode of action of these compounds to kill pathogens (Kettner and Dörffling, 1995; Malik et al., 2019). However, inadequate information is available about the interaction of these VOCs produced by *P. pseudoalcaligenes* SRM-16 against phytopathogen RS. Yasmin et al. (2021) reported that active VOCs produced by *P. pseudoalcaligenes* SRM-16 were 2-pentylfuran, 2,3-butanediol, and dimethyl disulfide. Docking analysis of other ligands has been reported in various studies against CRZ1 and S9 protein receptors of RS (Kettner and Dörffling, 1995; Malik et al., 2019). In this study, for the first time, docking analysis was performed to check the binding affinities of the above ligands with CRZ1 and S9 protein receptors of RS. Our results clearly indicate an overall binding trend of each conformation for VOCs 2-pentylfuran, 2,3-butanediol, and dimethyl disulfide. It was observed that the 2-pentylfuran ligand binds to the CRZ1 protein receptor at active binding pockets. In frames 3, 4, and 5, we found 2-pentylfuran ligand attachment at Ala648 residue, while in frame 9, we discovered ligand attachment at Leu635 and Gly595 residues. However, 2,3-butanediol and dimethyl disulfide were unable to bind at any of the active binding pockets of receptor CRZ1 in all of the nine frames. It has been reported that different antifungal metabolites showed binding potential at CRZ1 protein receptors (**Figure 8**) (Malik et al., 2019). Proteomics study has revealed that VOCs downregulate the proteins that are not only related to virulence but also to carbohydrate and amino acid metabolism, protein folding, translation, genetic information, cellular processes, and antioxidant activity. This can inhibit the growth and root colonization of pathogens. Microbial VOCs may also damage the DNA of pathogens (Almeida et al., 2023).

The remarkable findings of this study indicated that 2-pentylfuran exhibited binding affinity with both CRZ1 and S9 receptors of RS, while 2,3-butanediol and dimethyl disulfide exhibited binding affinities with only S9 protein. Hence, to restrict the development of RS, S9 protein receptors are more likely to be targeted by VOCs than CRZ1 receptors.

## Conclusion

Our results indicate that VOCs (2-pentylfuran, 2,3-butanediol, and dimethyl disulfide) released by *P. pseudoalcaligenes* SRM-16 have great potential to antagonize RS, as demonstrated by successful restriction of pathogen progression and increase in biomass in maize plants. Maize acquire systemic resistance by upregulating defense machinery followed by enhanced photosynthesis and increased activities of defense enzymes. The VOCs aided in the acquisition of systemic resistance. The inhibition potential of these VOCs exhibits variation with CRZ1 and S9 protein receptors of RS. We discovered that 2-pentylfuran had binding interactions with both the CRZ1 and S9 RS receptors, but 2,3-butanediol and dimethyl disulfide had binding affinity for only the S9 protein. Additional research could assist in the confirmation of these compounds as possible antifungal drugs. With our key findings, all of the compounds could be further explored for structural change in terms of developing potentially innovative and effective antifungal agents.

## Data availability statement

The original contributions presented in the study are included in the article/**Supplementary Material**. Further inquiries can be directed to the corresponding authors.

## Author contributions

HY, ZS, SM, and NI drafted the experimental design, and ZS, SM, and UR performed the experiments. AA and YC analyzed the data and revised the article to present form. All authors contributed to the article and approved the submitted version.
