# Supplementary material for: Alleviation of banded leaf and sheath blight disease incidence in maize by bacterial volatile organic compounds and molecular docking of targeted inhibitors in Rhizoctonia solani
Source: Front Plant Sci. 2023 Sep 13;14:1218615. doi: 10.3389/fpls.2023.1218615 (PMC10588623; doi:10.3389/fpls.2023.1218615)
Supplement: Supplementary file 1 [file DataSheet_1.docx]

**Table S1. Blind docking of 2, pentyl furan, 2, butanediol and dimethyl sulphide with CRZ1 protein receptor**

| **Ligands** | **Conformations** | **Binding Affinity** | **rmsd/ub** | **rmsd/lb** |
| --- | --- | --- | --- | --- |
| 2_pentylfuran_uff_E=219.63 | Fram1 | -4.3 | 0 | 0 |
| 2_pentylfuran_uff_E=219.63 | Fram2 | -4 | 69.286 | 67.85 |
| 2_pentylfuran_uff_E=219.63 | Fram3 | -4 | 70.813 | 69.411 |
| 2_pentylfuran_uff_E=219.63 | Fram4 | -3.9 | 69.454 | 68.121 |
| 2_pentylfuran_uff_E=219.63 | Fram5 | -3.9 | 3.758 | 1.517 |
| 2_pentylfuran_uff_E=219.63 | Fram6 | -3.8 | 39.023 | 37.978 |
| 2_pentylfuran_uff_E=219.63 | Fram7 | -3.8 | 69.265 | 67.946 |
| 2_pentylfuran_uff_E=219.63 | Fram8 | -3.8 | 24.79 | 24.18 |
| 2_pentylfuran_uff_E=219.63 | Fram9 | -3.8 | 3.972 | 1.744 |
| 3_butanediol_uff_E=56.06 | Fram1 | -2.8 | 0 | 0 |
| 3_butanediol_uff_E=56.06 | Fram2 | -2.7 | 2.511 | 1.87 |
| 3_butanediol_uff_E=56.06 | Fram3 | -2.7 | 2.86 | 1.672 |
| 3_butanediol_uff_E=56.06 | Fram4 | -2.7 | 2.33 | 1.708 |
| 3_butanediol_uff_E=56.06 | Fram5 | -2.7 | 3.051 | 1.761 |
| 3_butanediol_uff_E=56.06 | Fram6 | -2.7 | 2.871 | 1.342 |
| 3_butanediol_uff_E=56.06 | Fram7 | -2.6 | 4.029 | 3.22 |
| 3_butanediol_uff_E=56.06 | Fram8 | -2.5 | 9.277 | 8.495 |
| 3_butanediol_uff_E=56.06 | Fram9 | -2.4 | 7.813 | 7.446 |
| Dimethyldisulphide_uff_E=47.05 | Fram1 | -1.6 | 0 | 0 |
| Dimethyldisulphide_uff_E=47.05 | Fram2 | -1.5 | 2.854 | 0.868 |
| Dimethyldisulphide_uff_E=47.05 | Fram3 | -1.5 | 9.89 | 9.252 |
| Dimethyldisulphide_uff_E=47.05 | Fram4 | -1.5 | 1.913 | 1.761 |
| Dimethyldisulphide_uff_E=47.05 | Fram5 | -1.5 | 9.549 | 8.666 |
| Dimethyldisulphide_uff_E=47.05 | Fram6 | -1.5 | 2.101 | 1.81 |
| Dimethyldisulphide_uff_E=47.05 | Fram7 | -1.4 | 9.712 | 9.356 |
| Dimethyldisulphide_uff_E=47.05 | Fram8 | -1.3 | 3.851 | 3.355 |
| Dimethyldisulphide_uff_E=47.05 | Fram9 | -1.2 | 4.301 | 2.954 |

**Table S2. Blind docking of 2, pentyl furan, 2, butanediol, and dimethyl sulfide with an S9 protein receptor**

| **Ligand** | **Conformations** | | **Binding Affinity** | **rmsd/ub** | **rmsd/lb** |
| --- | --- | --- | --- | --- | --- |
| S9_model1_2_pentylfuran_uff_E=219.63 | | Fram1 | -4.1 | 0 | 0 |
| S9_model1_2_pentylfuran_uff_E=219.63 | | Fram2 | -4.1 | 14.398 | 13.254 |
| S9_model1_2_pentylfuran_uff_E=219.63 | | Fram3 | -4.1 | 14.625 | 13.897 |
| S9_model1_2_pentylfuran_uff_E=219.63 | | Fram4 | -4.1 | 14.142 | 13.229 |
| S9_model1_2_pentylfuran_uff_E=219.63 | | Fram5 | -4 | 11.472 | 9.528 |
| S9_model1_2_pentylfuran_uff_E=219.63 | | Fram6 | -4 | 15.213 | 13.767 |
| S9_model1_2_pentylfuran_uff_E=219.63 | | Fram7 | -4 | 14.645 | 13.352 |
| S9_model1_2_pentylfuran_uff_E=219.63 | | Fram8 | -3.9 | 14.739 | 13.736 |
| S9_model1_2_pentylfuran_uff_E=219.63 | | Fram9 | -3.9 | 14.589 | 13.205 |
| S9_model1_2_3_butanediol_uff_E=56.06 | | Fram1 | -3.2 | 0 | 0 |
| S9_model1_2_3_butanediol_uff_E=56.07 | | Fram2 | -3.2 | 22.297 | 21.482 |
| S9_model1_2_3_butanediol_uff_E=56.08 | | Fram3 | -3.1 | 2.948 | 0.317 |
| S9_model1_2_3_butanediol_uff_E=56.09 | | Fram4 | -3.1 | 22.1 | 21.358 |
| S9_model1_2_3_butanediol_uff_E=56.10 | | Fram5 | -3.1 | 23.442 | 22.686 |
| S9_model1_2_3_butanediol_uff_E=56.11 | | Fram6 | -3 | 23.568 | 22.72 |
| S9_model1_2_3_butanediol_uff_E=56.12 | | Fram7 | -3 | 29.037 | 28.333 |
| S9_model1_2_3_butanediol_uff_E=56.13 | | Fram8 | -3 | 29.045 | 28.336 |
| S9_model1_2_3_butanediol_uff_E=56.14 | | Fram9 | -2.9 | 21.939 | 21.161 |
| S9_model1_Dimethyldisulphide_uff_E=47.05 | | Fram1 | -2 | 0 | 0 |
| S9_model1_Dimethyldisulphide_uff_E=47.05 | | Fram2 | -2 | 3.373 | 0.264 |
| S9_model1_Dimethyldisulphide_uff_E=47.05 | | Fram3 | -1.9 | 3.359 | 2.683 |
| S9_model1_Dimethyldisulphide_uff_E=47.05 | | Fram4 | -1.9 | 2.698 | 2.247 |
| S9_model1_Dimethyldisulphide_uff_E=47.05 | | Fram5 | -1.9 | 2.965 | 2.684 |
| S9_model1_Dimethyldisulphide_uff_E=47.05 | | Fram6 | -1.7 | 11.304 | 10.923 |
| S9_model1_Dimethyldisulphide_uff_E=47.05 | | Fram7 | -1.7 | 28.033 | 27.711 |
| S9_model1_Dimethyldisulphide_uff_E=47.05 | | Fram8 | -1.6 | 11.693 | 10.871 |
| S9_model1_Dimethyldisulphide_uff_E=47.05 | | Fram9 | -1.5 | 11.928 | 11.315 |

**
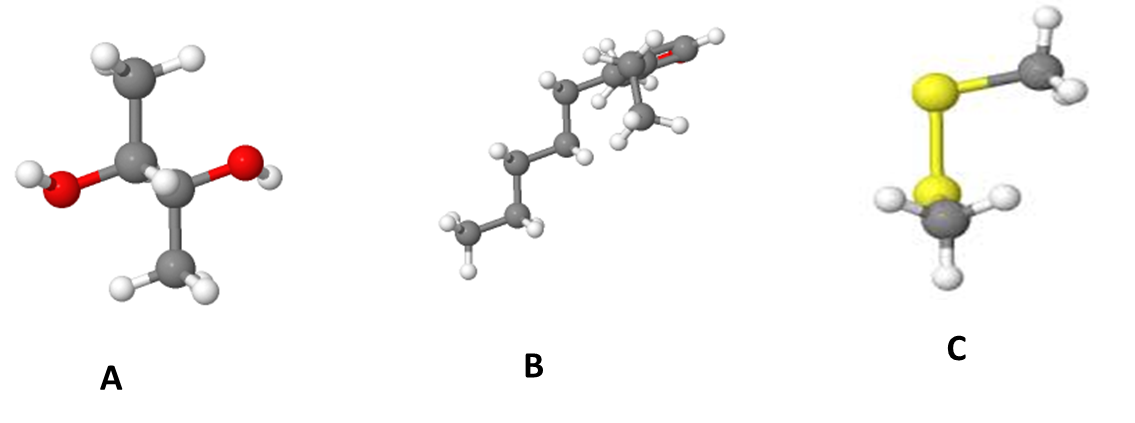
**

**Figure S1 3D structures of A) 2, 3 butanediol, B) Pentylfuran C) Dimethyl disulphide**
